# Prostate cancer infiltrating the bladder sphincter successfully treated with Electrochemotherapy: a case report

**DOI:** 10.1002/ccr3.1270

**Published:** 2017-11-13

**Authors:** Nina Klein, Enric Gunther, Stefan Zapf, Rachid El‐Idrissi, Johannes Atta, Michael Stehling

**Affiliations:** ^1^ Prostate Center Institut für Bildgebende Diagnostik Strahlenbergerstr. 110 63067 Offenbach am Main Germany

**Keywords:** Electrochemotherapy, minimally invasive, prostate cancer, urethral sphincter infiltration

## Abstract

We demonstrate feasibility and safety of Electrochemotherapy for treatment of a prostate cancer (PCa) with infiltration of the urethral sphincter. The patient remained continent and potent, toxicity was low, and 6 months of follow‐up showed no cancer activity. We conclude that Electrochemotherapy should be further evaluated as treatment strategy for locally advanced PCa.

## Background

Prostate cancer is a very common form of cancer worldwide, with millions of new cases every year 1. There are several ways to treat prostate cancer (PCa), the most established among them being radical prostatectomy and radiation therapy. PCa patients with cancer infiltration of surrounding tissue and organs, that is, rectal infiltration or infiltration of the lower urinary sphincter, can face severe adverse events when undergoing these treatments 2. Therefore, new alternative strategies are sought for that involve less toxic agents and a minimally invasive approach for continence‐ and potency‐preserving treatment of advanced PCa. Several focal therapies such as Irreversible Electroporation and Cryosurgery have previously been suggested and successfully applied 3, 4.

Electrochemotherapy (ECT), the combination of cell electroporation and the application of a chemotherapeutic agent, has advanced to be an acknowledged and well‐established nonthermal ablation modality in treatment of cutaneous and subcutaneous tumors of different histological origins. With the publication of the European Standard Operating Procedures of Electrochemotherapy (ESOPE) in 2006, the awareness of ECT increased especially for therapy of cutaneous tumors and metastases 5, but by now has also been reported feasible and safe for some deep‐seated tumors 6, also in combination with Irreversible Electroporation 7. Its low toxicity and high response rate are the main advantages of the therapy, but ECT is an attractive approach especially in cases where tumor size and/or location require a precise and gentle form of treatment to spare surrounding healthy cells, tissues and organs, due to its high specificity and low cumulative doses of chemotherapeutic drug 8.

Here, we present the first case of a patient with prostate cancer with infiltration of the external urethral sphincter, who was facing severe adverse events upon standard therapies. Electrochemotherapy served as a gentle alternative treatment approach to preserve the function of all surrounding organs, especially the directly affected sphincter. The patient tolerated the procedure well, and the cancer was successfully removed while the sphincter was fully preserved, showing no cancer activity at 6 months of follow‐up. The patient regained his pretreatment potency and continence stage. We therefore conclude that ECT of the prostate is feasible, and in this case study effective and safe, even when infiltration of surrounding tissue is involved.

## Case Presentation

### Clinical data

**Figure 1:**
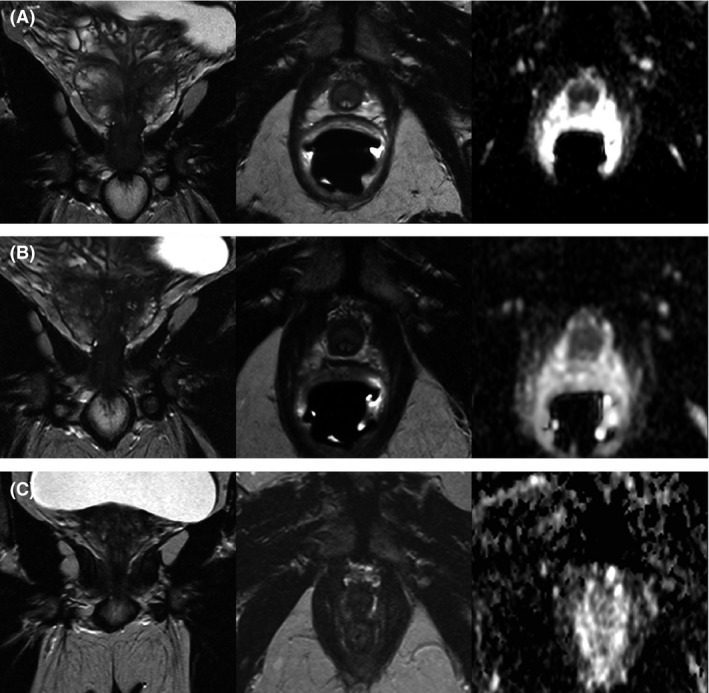
Preinterventional magnetic resonance images, showing t2 coronal (left), t2 axial (middle), and ADC‐map images (right) to calculate ADC values of the tumor site. MRI images in A and B were acquired with an endorectal coil to increase sensitivity. (A) Pre‐ADT therapy, ADC (measured in central lesion) approx. 568 ± 89 mm^2^/sec. (B) 4 weeks into AHD therapy, ADC approx. 739 ± 36 mm^2^/sec. (C) After three months of ADT and 1 day before intervention, ADC approx. 700 ± 145 mm^2^/sec. Images show the tumor growth along the urethra. Initial prostate volume was approx. 35 mL, but got reduced to 19 mL pretreatment. A detailed description is given in the text.

Sixty seven‐year‐old patient presented with biopsy‐proven recurrence of prostate carcinoma Gleason 6 and 7b in the apex of the prostate (PIRADS 6), clinical stage T4, with suspected tumor growth along the urethra. PSA was 7.1 ng/mL, with values fluctuating between 7 and 7.5 ng/mL. Patient had undergone TUR‐P 2 years before. Initial IPSS‐score was 2 of 35 (equivalent to mild incontinence) with no impairment in quality of life and IIEF‐5 score 21 of 25 (mild erectile dysfunction (ED)), respectively. Multiparametric magnetic resonance imaging (mpMRI) revealed a prostate volume of approx. 35 mL with a 0.4 × 1.0 cm large semicircular lesion in the left anterior glandular wall, in the transition of the anterior transition zone to the anterior stroma, as well as an approx. 1.2 × 1.5 × 2.4 cm large tumor formation medially, completely encompassing the intraprostatic urethra, which reached up to the corpus spongiosum in the caudal direction and no longer showed the bladder sphincter as an intrinsic structure (Fig. 1A). A simultaneous occurrence of an initial benign prostatic hyperplasia (BPH) of the transitional zone with inflammatory changes could be seen. Postinflammatory changes of the peripheral zone on both sides with left‐sided emphasis were detected. No evidence of pelvic lymphadenopathy or bony metastases was found.

The recommendation of androgen deprivation therapy (ADT) with Bicalutamide 150 was given, which the patient agreed to. After 4 weeks, a regress of the PCa in the apex and around the urethra was seen in MRI (Fig. 1B). No evidence for a simultaneous urothelial carcinoma could be found; however, BPH status and inflammatory changes were unchanged.

A treatment with radical prostatectomy or radiation therapy would have most likely resulted in incontinence and impotence due to the infiltration of the bladder sphincter and the corpus spongiosum. The treatment with ECT offered the possibility to at least partially preserve these functions. ECT was applied 6 weeks later, with continuation of the androgen deprivation therapy with the goal of further tumor reduction. mpMRI 1 day prior treatment illustrated a reduction in prostate volume to 19 mL and further regredience of the PCa lesions in the apex of the prostate (Fig. 1C).

### Intervention

**Figure 2:**
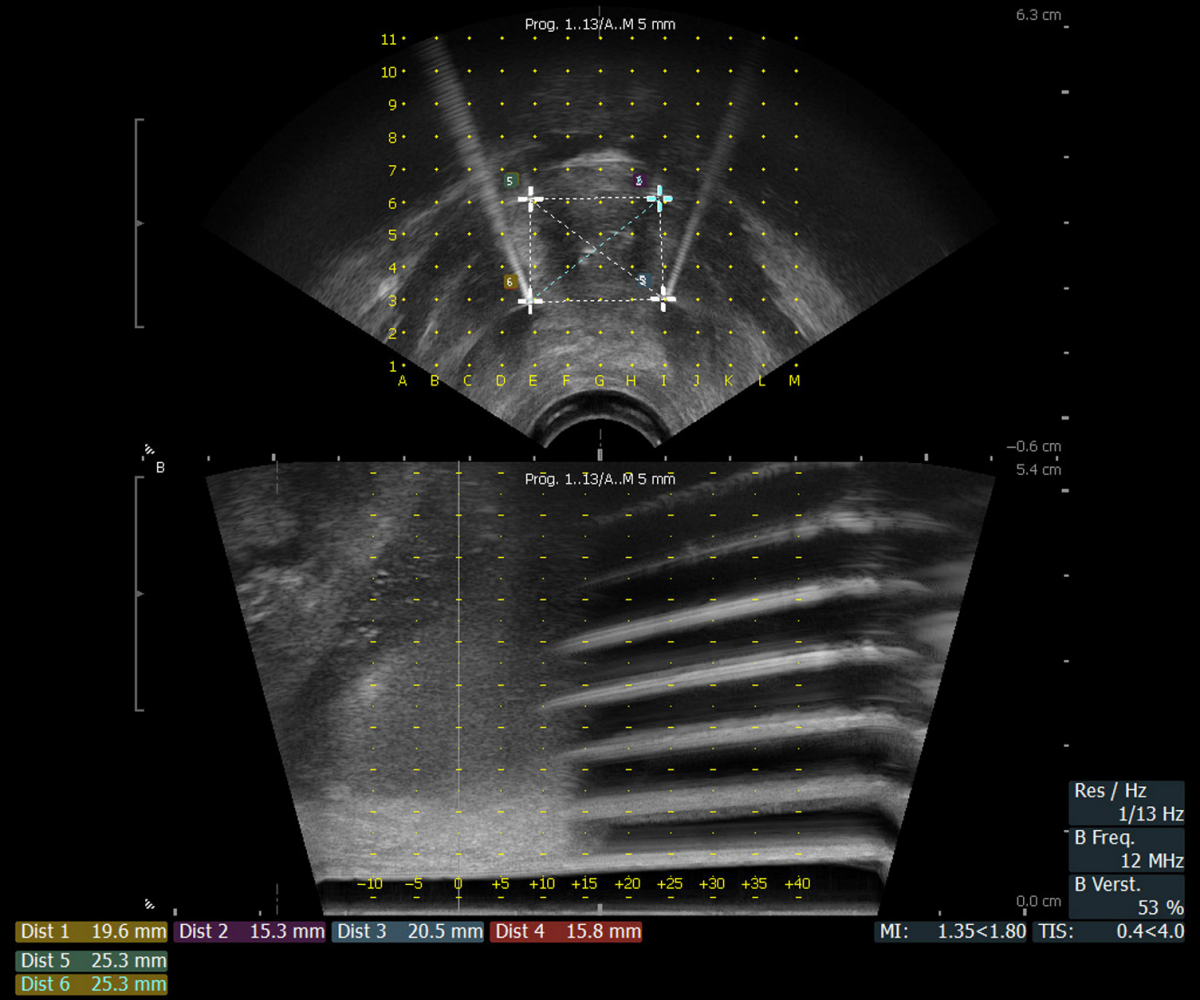
Axial (top) and sagittal (bottom) ultrasound images during the procedure, showing the electrode configuration for ECT treatment. Electrodes form a square, with distances between them which are suitable to form a homogenous ECT treatment field (distances are given at the bottom left).

Patient was positioned in lithotomy position, and a bladder catheter (CH18) was inserted. Treatment was carried out under general anesthesia and deep muscle relaxation. A total of four electrodes (Angiodynamics, Latham, NY) were then inserted percutaneously through the perineum under rectal ultrasound guidance (BK Ultrasound FlexFocus 400 with 8848 endorectal linear biplane transducer, Analogic Corporation, Peabody, MA) (Fig. 2), each in pairs ventrally and dorsally of the sphincter externus, arranged in a square (see Fig. 2 for dimensions), and connected to the generator output of the Cliniporator Vitae (IGEA, Carpi, Italy). These electrodes were used be able to adjust an exposure length to precisely 2.5 cm. The tumor volume was completely enclosed. Subsequent unproblematic ECT was followed, with administration of a total of 29 mg of Bleomycin i.v. 8 min prior to electroporation, according to ESOPE 5 with 8 100‐msec square pulses with a rise time of 1 msec and at a frequency of 4 Hz. Further necessary electrical parameters (according to 9) were as follows: mean voltage amplitude applied 1642 ± 812 V, mean measured current 21.6 ± 14.3 A. The total duration of the intervention was 80 min.

## Results

**Figure 3:**
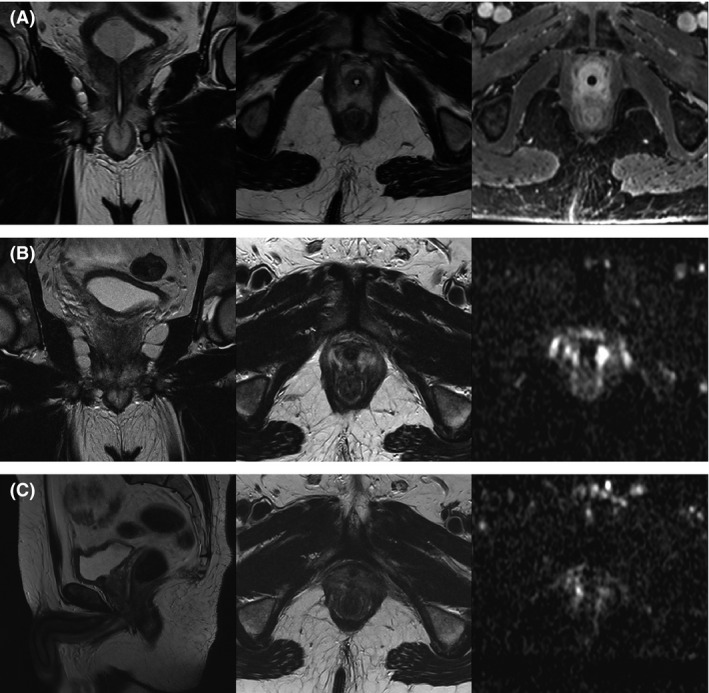
Postinterventional magnetic resonance images. (A) MRI 1 day post‐ECT showing t2 coronal (left), t2 axial (middle), and fl3d vibe (right), the latter illustrating the postcontrast agent state which is particularly useful for the simultaneous evaluation of soft tissue and vasculature. Mild edema, but no hematuria or bleeding can be seen. (B) MRI 8 weeks post‐ECT showing t2 coronal (left), t2 axial (middle), and ADC‐map images to calculate ADC values of the tumor site (right, ADC <100 mm^2^/sec). Treatment area shows avascularity without tumor persistence. (C) MRI 6 months post‐ECT showing sagittal (bottom left), t2 axial (middle), and ADC‐map images (ADC <100 mm^2^/sec). No tumor persistence, no pathologic contrast enhancement or diffusion restriction can be detected, and all surrounding tissues and organs are without tumor infiltration.

MRI 24 h post‐ECT showed normal postinterventional findings: mild edema of prostatic apex, no bleeding, no hematoma, and no encapsulated fluid collections (Fig. 3A). Catheter remained in situ and was kept for 12 days, which resulted in slight discomfort for the patient. Patient was further treated with Ciprobay 500 and Voltaren retard (Gastric protection with Pantoprazole). Follow‐up MRI 8 weeks after ECT showed a typical avascular lesion within the treated area without evidence of tumor persistence, no pathologic contrast enhancement could be detected (Fig. 3B). Patient completed the IPPS‐questionnaire with a total score of 5 of 35 (remaining with mild incontinence) and an IIEF‐5 score of 16 (mid‐ to moderate ED). Finally, 6 months after the treatment, another MRI was acquired, which showed uneventful postinterventional findings without evidence of tumor persistence or lymphadenopathy (Fig. 3C), with a very small‐volume prostate, unchanged from the preliminary examination. No surrounding infiltration was seen; bladder bottom, seminal vesicles, neurovascular bundle, urethra and external sphincter, and rectal wall were without indication of infiltration. Still no pathologic contrast agent uptake or pathological ADC values could be detected. The bladder showed itself inconspicuous, with no indication of secondary urinary back‐pressure. IPPS‐score was still at 5 (mild incontinence), mainly due to small amounts of urine leakage approx. once a day, while IIEF‐5 score increased to 19 (equivalent to mild ED), now restoring to the range pretreatment. PSA 6 months after ECT was 1 ng/mL. Patient will continue to have PSA screening every 3 months and MRI in 6 months.

## Discussion

Patients with extraprostatic disease (pathologic extension beyond the prostate gland, positive surgical margins, or invasion of the seminal vesicle) face a higher risk of disease recurrence, progression, and death than those with organ‐confined disease 10, 11, 12. The treatment of such cancers remains a challenge, as most available treatment options apprehend the danger of tissue or organ damage, which may result in major adverse events such as incontinence or impotency. In such cases, a gentle yet effective approach is favorable to perform tumor mass reduction without impairment of the infiltrated structures. Several focal therapies have been suggested as a gentle alternative for treatment of PCa, among them Irreversible Electroporation and Cryosurgery 3, 4, although so far the main focus was on organ‐confined PCa for the application of these focal therapies.

Electrochemotherapy is a minimally invasive alternative to focally treat cancerous tissue with substantial reduction in adverse events. It has been successfully applied in cutaneous and subcutaneous tumors of different histological origins, and is now approaching as an option for deep‐seated tumors. Its gentle approach makes it possible to treat tumors even in close proximity to large blood vessels 13. We present the first case of a successful ECT to treat prostate cancer. In our case, the patient had locally advanced PCa with infiltration of the bladder sphincter. The patient, who also suffered from benign prostatic hyperplasia, started androgen deprivation therapy three months prior treatment to reduce prostate volume (Fig. 1). ECT was applied in a single session with a total Bleomycin dosage of 29 mg.

Treatment was without any major side effects; no adverse events resulting from Bleomycin administration or the electroporation procedure could be reported. The treatment was overall well tolerated; only a slight discomfort due to the catheter was noted, which had to be carried for 12 days. The feeling of discomfort vanished after catheter removal. IPSS‐evaluation revealed no significant impairment (remaining at mild incontinence with minor urge incontinence), while IIEF5‐score was almost restored to values pretreatment after 6 months. This is a remarkable result, as the urethra was included within the treatment field, and transient incontinence could have been a possible adverse event. However, no urethra sphincter impairment could be detected (Fig. 3). All of these points illustrate the overall tolerability of the treatment and its low toxicity to surrounding structures.

Abdominal MRI 24 h after intervention showed mild edema within the treatment area, while no bleeding, no hematoma, and no fluid/air collections could be found (Fig. 3A). Follow‐up MRI 8 weeks and 6 months after ECT was completely uneventful without evidence of any tumor activity (Fig. 3B and C). PSA values went from 7 ng/mL pretreatment to 1 ng/mL 6 months after treatment; this is expected, as PSA values will not go below detection rate when PCa is treated focally and healthy prostate tissue remains. These findings indicate efficacy and antitumor activity of the treatment within a follow‐up time of 6 months.

The transperineal access for electrode placement (Fig. 2) made it possible to treat the patient without surgical intervention. This minimally invasive approach certainly contributed to the limited occurrence of adverse events and had the advantage of reducing the hospital stay to a single day (outpatient).

The treatment itself was feasible to perform; however, as with all minimally invasive focal therapeutic approaches that require precise treatment planning and good‐quality diagnostic imaging, it is crucial to locate the cancer as accurately as possible in order to succeed in tumor ablation.

To further investigate the efficacy of ECT in locally advanced prostate cancer, a clinical trial with several patients and a longer follow‐up time is warranted to address the impact of this treatment strategy on recurrence‐free survival. Nevertheless, this case study was able to demonstrate that ECT was a feasible and safe option for the presented patient with PCa that has infiltrated the bladder sphincter, who remained continent and potent after treatment.

## Conclusion

This case report describes the successful Electrochemotherapy (ECT) of a patient with prostate cancer with infiltration of the urethral sphincter. Toxicity of the treatment was low, with only mild adverse events (mild hematuria of the prostate, slight transient urge incontinence, and discomfort due to catheterization). The patient had fully restored IIEF5 and IPSS‐scores 6 months after the treatment, remaining within the prior scoring range equivalent to mild incontinence and mild erectile dysfunction. Magnetic resonance images 6 months post‐ECT show no sign of tumor activity or impairment in the surrounding tissue or organs. These findings indicate that ECT of the prostate is a safe and feasible treatment option and can even be performed in cases of patients with PCas who are not organ‐confined. We therefore purpose further clinical investigation to assess data on long‐term recurrent‐free survival to evaluate ECT as a general approach for treatment of patients with locally advanced PCa.

## Authorship

NK, SZ, EG, MKS: contributed to treatment planning and execution, and the preparation, review, and submission of the manuscript. JA: reviewed the manuscript.

## Conflict of Interest

The authors report no relevant conflict of interests.
